# Antibacterial Effect of Hydroalcoholic Extract of* Punica granatum* Linn. Petal on Common Oral Microorganisms

**DOI:** 10.1155/2016/8098943

**Published:** 2016-01-14

**Authors:** Farnaz Hajifattahi, Elham Moravej-Salehi, Maryam Taheri, Arash Mahboubi, Mohammad Kamalinejad

**Affiliations:** ^1^Department of Oral Medicine, Islamic Azad University, Dental Branch, Tehran 19486, Iran; ^2^Department of Operative Dentistry, School of Dentistry, Shahed University, Tehran, Iran; ^3^Private Practice, Tehran, Iran; ^4^Department of Pharmaceutics, School of Pharmacy, Shahid Beheshti University of Medical Sciences, Tehran, Iran; ^5^Department of Pharmacognosy, School of Pharmacy, Shahid Beheshti University of Medical Sciences, Tehran, Iran

## Abstract

*Objectives*. This study aimed to assess the effect of hydroalcoholic extract of* Punica granatum* Linn. (*P. granatum*) petal on* Streptococcus sanguinis*,* Streptococcus mutans*,* Streptococcus salivarius*,* Streptococcus sobrinus,* and* Enterococcus faecalis*.* Materials and Methods*. In this in vitro study,* P. granatum* extract was prepared using powdered petals and water-ethanol solvent. Antibacterial effect of the extract, chlorhexidine (CHX), and ampicillin was evaluated on brain heart infusion agar (BHIA) using the cup-plate method. By assessing the diameter of the growth inhibition zone, the minimum inhibitory concentration (MIC) and minimum bactericidal concentration (MBC) of the extract were determined for the above-mentioned bacteria.* Results*. Hydroalcoholic extract of* P. granatum* petal had inhibitory effects on the proliferation of all five bacterial strains with maximum effect on* S. mutans* with MIC and MBC of 3.9 mg/mL. The largest growth inhibition zone diameter belonged to* S. sanguinis* and the smallest to* E. faecalis*. Ampicillin and CHX had the greatest inhibitory effect on* S. sanguinis*.* Conclusions*. Hydroalcoholic extract of* P. granatum* had a significant antibacterial effect on common oral bacterial pathogens with maximum effect on* S. mutans*, which is the main microorganism responsible for dental plaque and caries.

## 1. Introduction

Dental caries, periodontal disease, and opportunistic infections are among the common diseases of the oral cavity caused by the accumulation of pathogenic microorganisms, inappropriate nutritional habits, and improper oral hygiene [1–4]. Oral streptococci are the first isolated species to play a role in the formation of dental plaque and development of caries [5–7]. Measures taken to clinically inhibit plaque accumulation, namely, tooth brushing, dental flossing, and use of mouth rinses (as an adjunct), are highly effective for preventing the supragingival plaque and gingivitis and decreasing the number of microorganisms [8]. Chlorhexidine is among the most widely used antimicrobial mouthwashes. However, despite extensive antimicrobial activity, it has side effects such as altered sense of taste and staining of tooth surfaces and restorations [9–11].

Herbal medicine has a long history. Many individuals residing in developing countries strongly believe in the efficacy of herbal medications for primary care [12, 13]. Considering the increasing resistance of microorganisms to chemical synthetic drugs and their side effects, researchers are becoming more and more interested in finding alternative herbal medications and their active components [14–18].

The pomegranate with the botanical name of* Punica granatum* Linn. is a fruit-bearing deciduous shrub or small Asian tree from the family of Punicaceae growing between 5 to 8 meters tall. It is native to Iran and northern India and has long been cultivated in the Asian Mediterranean region, Europe, and Africa.* Punica granatum* is rich in bioactive compounds, which are used for treatment of cancer, cardiovascular diseases, diabetes mellitus, dental diseases, bacterial infection, antibiotic resistance, skin conditions due to UV radiation, diarrhea, bloody diarrhea, and hemorrhoids. Moreover,* P. granatum* is used as a mouth rinse for treatment of some types of sore throat. Parts of the plant used for medicinal purposes include its flowers, trunk skin, fruits, roots, and seeds [19–21].

The* Punica granatum* flowers are odorless but colorful red or reddish, 3.5 to 7 cm in length, and campanulate or cylindrical. Flowers are two types: fertilized and unfertilized. The unfertilized flower is with smaller, barren, and short-styled, short-stamened petals, in which the stigma is far below the anthers. The unfertilized flowers are commonly known as “Golnar” in Iranian traditional and complementary medicine [22].

Trunk skin and roots are used for parasitic infections, dried flowers are beneficial for treatment of bronchitis, diarrhea, and bloody diarrhea, and the brewed pomegranate is used for treatment of inflammation of the throat and oral cavity. Pomegranate has long been recommended as a hemostatic agent and for treatment of diabetes mellitus in ancient Greek medicine [19–21].

The antimicrobial properties of* P. granatum* have been recently noticed [19, 23–30]. The ethanol, water, methanol, and acetone extracts of* P. granatum* have shown strong antimicrobial properties against Gram-positive and Gram-negative nonoral microorganisms [14, 19, 22, 24, 31]. However, a few studies have evaluated the antibacterial properties of this plant on oral bacteria [14, 31–33].

However, the mentioned studies are very limited and mostly lack an appropriate microbiological method. Their methodology is often not clear or not well described. Search of the literature only yielded one previous study on the effect of water extract of* P. granatum* flower (petal) on oral microbial pathogens, which showed its greatest antimicrobial effect on* S. sanguinis* [31], although Menezes et al. reported that hydroalcoholic extract of* P. granatum* was very effective against biofilm forming microorganisms in the mouth of patients [34].

Considering the high prevalence of oral and dental diseases due to oral pathogens and the recent public interest in medicinal plants, this study aimed to assess the effect of hydroalcoholic extract of* P. granatum* petal on* S. mutans*,* S. sanguinis*,* S. salivarius, S. sobrinus,* and* E. faecalis* in vitro.

## 2. Materials and Methods

*P. granatum* flowers were obtained from the last harvest in Darab city and its purity was confirmed in a pharmacognosy laboratory.* P. granatum* extract was prepared by maceration method. Powdered petals were precisely measured by a digital scale and poured into an Erlenmeyer flask. Hydroalcoholic solvent (50% water and 50% ethanol) was also added. The Erlenmeyer flasks were capped with aluminum foil and stored in the dark for 10 days. Next, the flasks were placed on a shaker (GFL 3017) operating at 90 rpm for 24 hours. The solutions were then paper filtered. The filtered solution was poured into a sterile glass container and capped by aluminum foil. A few holes were perforated in the foil and the glass container was placed in Bain-Marie at 90°C to dry. The dried extract was precisely weighed, labeled, and refrigerated [14].

### 2.1. Activation of Microorganisms

Standard strains of* S. mutans* (ATCC 35668, PTCC 1683),* S. sanguinis* (ATCC 10556, PTCC 1449),* S. salivarius* (ATCC 9222, PTCC 1448),* S. sobrinus* (ATCC 27607 and PTCC 1601), and* E. faecalis* (ATCC 11700, PTCC 1393) were obtained in lyophilized form from the Persian Type Culture Collection center. Bacteria were activated by inoculation in the brain heart infusion agar (BHIA, Merck, Germany) culture medium followed by 24 hours of incubation at 37°C. For preparation of microbial suspension, a 24-hour culture was used. The concentration of microorganisms in the microbial suspension was adjusted to 0.5 McFarland standard using a spectrophotometer at a wavelength of 625 nm (a McFarland standard is a chemical solution with a turbidity comparable to that of microbial suspension. Using this suspension, number of bacteria per each milliliter of the suspension can be estimated, which is equal to 1.5 × 10^8^ CFU/mL) [35].

### 2.2. Assessment of Antimicrobial Effects

Primary assessment of the antimicrobial effect of extracts was done using the cup-plate technique. 500 *μ*L of each microbial suspension at 0.5 McFarland standard concentration was cultured in BHIA (swabbed on the plate). Then, wells measuring 8 mm in diameter were created on the agar surface. Different concentrations of the extracts were prepared by serial dilution (dilution by one-half) using sterile distilled water solvent; 100 *μ*L of each concentration of extract was poured into each well. The plates were incubated at 37°C (Memmert, Germany) for 24 hours. The diameter of the growth inhibition zone was measured in millimeters. This process was repeated in triplicate and the mean diameter of the growth inhibition zone was calculated for different concentrations of the extract [35].

### 2.3. Minimum Inhibitory Concentration

The MIC is defined as the lowest concentration of an antimicrobial agent that inhibits the growth of a microorganism (0.5 McFarland standard in this study). It is the minimum concentration of the extract that completely prevents visible growth and proliferation of bacteria compared to the negative control group. To determine MIC, macrodilution method according to the standard technique described by the clinical and laboratory standards institute (2012) was used. Different concentrations of the extract were prepared by serial dilution (dilution by one-half) in BHI broth medium. Using this medium, the 0.5 McFarland standard suspension was diluted 1 to 150 to obtain a bacterial count of 10^6^ CFU/mL. Microbial suspension was then diluted by one-half using the culture medium and 1 mL of it was added to the tubes containing serially diluted extract. The negative control tube only contained the culture medium and extract with no microbial suspension. The positive control tube contained culture medium and microbial suspension with no extract. After 24 hours of incubation at 37°C, growth and proliferation of microorganisms were evaluated and the MIC value of the extract for each bacterial strain was determined. This test was repeated in triplicate for each microorganism [35].

### 2.4. Minimum Bactericidal Concentration

After determination of MIC, 20 *μ*L of the suspension in the tube containing MIC of the extract and tubes showing no bacterial growth were cultured on plates containing BHIA. After 24 hours of incubation at 37°C, the plates were evaluated for growth of microorganisms. The concentration with no bacterial growth was determined as MBC. This test was repeated in triplicate for each microorganism [35].

The effect of 0.2% CHX (Shahr Daru, Iran) and ampicillin on the microorganisms was also evaluated using the cup-plate method and the MIC and MBC values of CHX and ampicillin for the microorganisms were determined as well.

### 2.5. Statistical Analysis

The tests were repeated in triplicate and the mean and standard deviation (SD) of the growth inhibition zone diameter in cup-plate method as well as the MIC and MBC of the extract, CHX, and ampicillin were determined.

## 3. Results

The hydroalcoholic extract of* P. granatum* showed inhibitory effects on the growth and proliferation of all five bacteria using the cup-plate method. The largest and the smallest diameter of growth inhibition zone belonged to* S. sanguinis* and* E. faecalis*, respectively. The mean (and SD) diameter of the growth inhibition zone due to the effect of hydroalcoholic extract of* P. granatum* on different microorganisms is shown in Table 1.

The MIC and MBC of hydroalcoholic extract of* P. granatum* were determined using serial dilution method. The highest antibacterial effect of* P. granatum* extract was on* S. mutans* with MIC and MBC values of 3.9 mg/mL. The MIC and MBC values of this extract for different microorganisms are presented in Tables 2 and 3.

The cup-plate method showed that 0.2% CHX had antimicrobial effect on all five bacterial strains. The mean and SD of the diameter of growth inhibition zone due to the effect of 0.2% CHX on the five bacterial strains are shown in Table 4.

Assessment of the MIC and MBC values revealed that CHX had the highest effect on* S. sanguinis*. Tables 2 and 3 show the MIC and MBC of CHX for the five bacterial strains.

The cup-plate method showed that ampicillin had inhibitory effects on the growth and proliferation of all five bacterial strains. The greatest diameter of growth inhibition zone belonged to* S. mutans* and* S. sanguinis* due to the effect of ampicillin. The mean and SD of the diameter of growth inhibition zone due to the effect of ampicillin on different microorganisms are shown in Table 5.

Assessment of the MIC and MBC values revealed that ampicillin had the highest effect on* S. sanguinis*. Tables 2 and 3 show the MIC and MBC of ampicillin for the five bacterial strains.

## 4. Discussion

This study was the first to experimentally assess the antimicrobial effect of hydroalcoholic extract of* P. granatum *petal on* S. mutans*,* S. sanguinis*,* S. salivarius*,* S. sobrinus,* and* E. faecalis* in vitro using the cup-plate method. The MIC, MBC, and growth inhibition zone diameter values of the extract for different microorganisms were also calculated and compared to CHX and ampicillin.

Based on the results, the hydroalcoholic extract of* P. granatum* petal had inhibitory effect on the growth and proliferation of all five bacterial strains. Vasconcelos et al. evaluated the MIC of pomegranate fruit gel and compared it with miconazole against adhesion of* S. mutans*,* S. mitis,* and* C. albicans*. They showed that pomegranate fruit gel was more effective than miconazole in preventing the adhesion of streptococci to glass [25]. Dahham et al. assessed the antimicrobial effect of ethanolic extract of seed, fruit, peel, and juice of pomegranate on specific bacteria and reported that the pomegranate peel extract had the greatest antimicrobial activity [23]. However, Abdollahzadeh et al. stated that only at concentrations of 8 mg/mL and 12 mg/mL methanolic extract of* P. granatum* peel (MEPGP) was effective against* L. acidophilus*,* S. mutans, *and* S. salivarius*. Furthermore, no concentrations of MEPGP inhibited* A. viscosus* and* C. albicans* [36]. In present study hydroalcoholic extract of* P. granatum* petal was effective against* S. mutans* in lower concentration than MEPGP in Abdollahzadeh et al. study. Therefore, it seems that hydroalcoholic extract of petal against* S. mutans* was more effective than MEPGP. It should be considered that* S. mutans* is main microorganism of dental plaque and caries [37]. Also, in current study, both MIC and MBC were considered. Furthermore, both MIC and MBC values against* S. mutans* were similar (3.9 mg/mL).

The MIC and MBC values of* P. granatum* showed the best antibacterial effect of this extract on* S. mutans*, while the lowest effect was noticed on* E. faecalis*. The only previous study on the antimicrobial effect of methanolic extract* P. granatum* flower on* S. mutans* was conducted by Haghighati et al. [32]. Their results showed no growth inhibition zone for* S. mutans*. Also, they showed no significant difference between the efficacy of* P. granatum* and CHX against* S. mutans*; these findings show that the method used was not proper and therefore their results are not reliable.

In a study by Vahid-Dastjerdi et al., the effect of water extract of* P. granatum* flower (petal) on the same microorganisms as in our study was evaluated. The highest antimicrobial effect was seen on* S. sanguinis* [31] compared to the MIC and MBC values obtained in the current study. Therefore, it appears that the solvent containing alcohol releases more amount of the antimicrobial agent and subsequently exerts a greater antimicrobial effect on* S. mutans*.

One important factor affecting the MIC is the difference in the composition of extracts. The composition of extract is influenced by the geographical location of the plant, season of harvesting, age of plant, growth stage, method of drying, and extraction technique. Also, extracts of different parts of the plant have variable level of antimicrobial activity and bacteria have variable sensitivity to different extracts. Also, isolated components of an extract show greater antimicrobial effects than the extract itself [38]. Alcoholic extract of *P. granatum* has well known isolated components such as gamma terpinene, borneol, camphor, carvacrol methyl ether, and methyl palmitate [39].

Camphor is one of the main components with antibacterial effect. A previous study isolated the constituents of rosemary and evaluated their effects on oral pathogenic microorganisms. Camphor had inhibitory effects on* S. mutans*,* S. sanguinis*,* S. salivarius*,* S. sobrinus,* and* E. faecalis* [40]. Carvacrol methyl ether is another constituent of* P. granatum* with confirmed antimicrobial effects. Thymol is an isomer of carvacrol, showing its antimicrobial activity by making the bacterial membrane permeable [39]. Chlorhexidine mouthwash has extensive antimicrobial activity and is more effective on Gram-positive than Gram-negative bacteria. It is known as the most effective mouthwash and the gold standard of antibacterial activity [41]. The MIC and MBC values and inhibitory effects obtained in our study for CHX were in accord with the results of specific microbiological assessments on CHX and ampicillin, which confirmed the accuracy and precision of the laboratory phases in our study.

One limitation of our study was that we only evaluated the* P. granatum* flowers harvested at a specific time from a specific location. Also, standardized microbial strains were used and microorganisms collected and purified from the oral cavity were not assessed. Thus, the results were limited to standard strains in the laboratory setting. Future studies are required to confirm the results in normal biological environment (oral cavity). Also, future studies must focus on the isolated constituents of different extracts and the antimicrobial agent in their composition. Given that the effects of these constituents on other microorganisms such as* L. acidophilus*,* A. viscosus*, and* C. albicans* are confirmed, they may be used in the form of pure extract, mouth rinse, or other antimicrobial products in clinical trials.

## 5. Conclusion

Within the limitations of this study, the following conclusions were drawn:

1. Hydroalcoholic extract of* P. granatum *had a significant antibacterial effect on common oral bacteria, namely,* S. sanguinis*,* S. mutans*,* S. salivarius*,* S. sobrinus,* and* E. faecalis*.
2. Hydroalcoholic extract of* P. granatum* had maximum antibacterial effect on* S. mutans* with MIC and MBC of 3.9 mg/mL.

## Figures and Tables

**Table 1:** The mean and SD of the diameter of growth inhibition zone (mm) due to the effect of the hydroalcoholic extract of *P. granatum* petal on different microorganisms.

Concentration (mg/mL)	Microorganism				
	*S. mutans*	*S. sanguinis*	*S. salivarius*	*S. sobrinus*	*E. faecalis*
125	17.5 ± 0.41	21.5 ± 0.41	16 ± 0	22.5 ± 0.41	15.5 ± 0.41
62.5	15 ± 0	20.5 ± 0.41	14 ± 0	20.5 ± 0.41	13 ± 0
31.25	12 ± 0	18.5 ± 0.41	12 ± 0	17.5 ± 0.41	10.5 ± 0.41
15.62	9.5 ± 0.41	18 ± 0	N.S.	15 ± 0.8	N.S.
7.81	N.S.	17 ± 0	N.S.	12 ± 0.8	N.S.
3.9	N.S.	15.5 ± 0.41	N.S.	N.S.	N.S.
1.95	N.S.	14.5 ± 0.41	N.S.	N.S.	N.S.

^**∗**^N.S.: not seen.

**Table 2:** The MIC (mg/mL) of hydroalcoholic extract of *P. granatum* petal, CHX, and Ampicillin for different microorganisms.

Microorganism	Substance		
	*P. granatum*	CHX	Ampicillin
*S. mutans*	3.9	0.09	0.06
*S. sanguinis*	7.81	0.02	0.015
*S. salivarius*	31.25	0.78	0.125
*S. sobrinus*	7.81	0.04	0.125
*E. faecalis*	125	6.25	2

**Table 3:** The MBC (mg/mL) of hydroalcoholic extract of *P. granatum* petal, CHX, and Ampicillin for different microorganisms.

Microorganism	Substance		
	*P. granatum*	CHX	Ampicillin
*S. mutans*	3.9	0.09	0.125
*S. sanguinis*	31.25	0.012	0.03
*S. salivarius*	62.5	0.39	0.125
*S. sobrinus*	31.25	0.02	0.25
*E. faecalis*	250	3.125	4

**Table 4:** The mean and SD of the diameter of growth inhibition zone (mm) due to the effect of 0.2% CHX on the five bacterial strains.

Concentration (*μ*g/mL)	Microorganism				
	*S. mutans*	*S. sanguinis*	*S. salivarius*	*S. sobrinus*	*E. faecalis*
100	>30 ± 0	>30 ± 0	>30 ± 0	>30 ± 0	23.5 ± 0.41
50	>30 ± 0	>30 ± 0	>30 ± 0	>30 ± 0	21 ± 0
25	>30 ± 0	>30 ± 0	>30 ± 0	>30 ± 0	18.5 ± 0.41
12.5	>30 ± 0	>30 ± 0	>30 ± 0	>30 ± 0	16.5 ± 0.41
6.25	>30 ± 0	>30 ± 0	>30 ± 0	>30 ± 0	14 ± 0
3.125	>30 ± 0	>30 ± 0	>30 ± 0	>30 ± 0	12 ± 0

**Table 5:** The mean and SD of the diameter of growth inhibition zone due to the effect of ampicillin on different microorganisms.

Concentration (*μ*g/mL)	Microorganism				
	*S. mutans*	*S. sanguinis*	*S. salivarius*	*S. sobrinus*	*E. faecalis*
16	>30 ± 0	>30 ± 0	>30 ± 0	>30 ± 0	18 ± 0
8	>30 ± 0	>30 ± 0	26.5 ± 0.61	28 ± 0	16 ± 0
4	30 ± 0	30.5 ± 0.41	22.5 ± 0.41	24.5 ± 0.41	14 ± 0
2	24.5 ± 0.41	25 ± 0	18 ± 0	16 ± 0	11.5 ± 0.41
1	18 ± 0	20 ± 0	15 ± 0	12 ± 0	N.S.
0.5	14.5 ± 0.41	16 ± 0	N.S.	N.S.	N.S.

^*∗*^N.S.: not seen.
